# *MyD88-*, but Not *Nod1*- and/or *Nod2*-Deficient Mice, Show Increased Susceptibility to Polymicrobial Sepsis due to Impaired Local Inflammatory Response

**DOI:** 10.1371/journal.pone.0103734

**Published:** 2014-08-01

**Authors:** Fabiane Sônego, Fernanda V. S. Castanheira, Paula G. Czaikoski, Alexandre Kanashiro, Fabricio O. Souto, Rafael O. França, Daniele C. Nascimento, Andressa Freitas, Fernando Spiller, Larissa D. Cunha, Dario S. Zamboni, José C. Alves-Filho, Fernando Q. Cunha

**Affiliations:** 1 Faculdade de Medicina de Ribeiraő Preto, Departamento de Farmacologia, Universidade de São Paulo, Ribeiraő Preto, São Paulo, Brasil; 2 Faculdade de Medicina de Ribeiraő Preto, Departamento de Biologia Celular e Molecular e Bioagentes Patogênicos, Universidade de São Paulo, Ribeiraő Preto, São Paulo, Brasil; McGill University, Canada

## Abstract

Pathogen recognition and triggering of the inflammatory response following infection in mammals depend mainly on Toll-like and Nod-like receptors. Here, we evaluated the role of Nod1, Nod2 and MyD88-dependent signaling in the chemokine production and neutrophil recruitment to the infectious site during sepsis induced by cecal ligation and puncture (CLP) in C57Bl/6 mice. We demonstrate that Nod1 and Nod2 are not involved in the release of chemokines and recruitment of neutrophils to the infectious site during CLP-induced septic peritonitis because these events were similar in wild-type, *Nod1*-, *Nod2*-, *Nod1/Nod2*- and *Rip2*-deficient mice. Consequently, the local and systemic bacterial loads were not altered. Accordingly, neither Nod1 nor Nod2 was involved in the production of the circulating cytokines and in the accumulation of leukocytes in the lungs. By contrast, we showed that MyD88-dependent signaling is crucial for the establishment of the local inflammatory response during CLP-induced sepsis. *MyD88*-deficient mice were susceptible to sepsis because of an impaired local production of chemokines and defective neutrophil recruitment to the infection site. Altogether, these data show that Nod1, Nod2 and Rip2 are not required for local chemokine production and neutrophil recruitment during CLP-induced sepsis, and they reinforce the importance of MyD88-dependent signaling for initiation of a protective host response.

## Introduction

Sepsis is a complex syndrome caused by the inability of the host to control an infection, typically by bacterial origin [1]. Sepsis is one of the most common causes of death in healthcare facilities worldwide, but a complete understanding of the physiopathology of this condition is still lacking [2].

The successful clearance of bacterial pathogens during sepsis has been clearly demonstrated to be dependent on efficient neutrophil recruitment to the infection site. To enable this process, host immune cells play a crucial role in establishing the local inflammatory response after detecting the pathogen [3]. Bacterial components are primarily detected by two families of pattern recognition receptors (PRR) in the host immune cell: Toll-like receptors (TLR) and Nucleotide-binding oligomerization domain (Nod)-like receptors (NLR) [4].

TLRs recognize a wide range of ligands from both Gram-positive and Gram-negative bacteria [5]. We have previously demonstrated that TLR2, TLR4 and TLR9 play a deleterious role in polymicrobial sepsis [6]–[8]. However, the beneficial or deleterious role of the adaptor protein MyD88, recruited by these TLRs, to the outcome of polymicrobial sepsis still remains controversial [9]–[13].

The prototypic receptors for the NLR family are Nod1 and Nod2, and these receptors sense the ligands iEDAP and muramyldipeptide (MDP), respectively. Both of the ligands are found in Gram-negative and Gram-positive bacterial cell walls. The downstream signaling from these receptors involves the adaptor protein Rip2 and the transcription factor NF-κB [14], [15]. Interestingly, *Nod2* polymorphisms have been associated with an increased susceptibility to sepsis in infant and adult patients [16]. Moreover, Nod1 ligands are able to induce chemokine production and neutrophil recruitment, and play an important role in the induction of organ dysfunction and shock [17], [18].

This study aimed to evaluate the role of the NLR family members Nod1 and Nod2 in the chemokines production and neutrophil recruitment to the infectious site in polymicrobial sepsis. Additionally, we addressed the role of MyD88 in the onset of sepsis. For enable these observation, we induced sepsis by the gold-standard cecal ligation and puncture (CLP) [19] and bacterial inoculation models. Here, we demonstrate that wild-type, *Nod1-, Nod2-, Nod1/Nod2-* and *Rip2*-deficient mice displayed similar levels of local chemokines, neutrophil recruitment to the infection site, local and systemic bacterial loads and survival. By contrast, the establishment of the local inflammatory response, which leads to the control of infection in polymicrobial sepsis, is triggered by MyD88-dependent signaling. *MyD88*-deficient mice were more susceptible to CLP-induced sepsis than wild-type mice. There was a large reduction in the levels of local inflammatory mediators and recruitment of neutrophils to the infection site in *MyD88*-deficient compared to wild-type mice. Furthermore, resident peritoneal cells that were adoptively transferred from wild-type mice to *MyD88*-deficient mice increased the chemokine production and neutrophil recruitment to the peritoneal cavity. Altogether, these data suggest that TLRs, but not Nod1 and Nod2, play a major role in the resolution of sepsis due to the establishment of the local response in polymicrobial sepsis.

## Materials and Methods

### Animals

Specific pathogen free (SPF) male and female C57Bl/6 (wild-type-WT) mice (weight 18–23 g) and *Tlr2*-, *MyD88*-deficient and *Tlr4*-mutant mice were obtained from Jackson Laboratory (Bar Harbor, USA). *Nod1*-, *Nod2*- and *Rip2*-deficient mice were kindly provided by Richard Flavell (Yale University). All of the mouse strains were bred in our institutional animal facilities. Mice doubly deficient for *Nod1* and *Nod2* were constructed by intercrossing the F1 generation and screening the F2 progeny for double deficiency in both genes by standard PCR procedures. Primers used for the screening were as follows: *Nod1* wild-type allele forward 5′GCTTGGCTCCTTTGTCATTG3′ and reverse 5′ACTGCTGCTTGGCTTTATTCTC3′; *Nod1* mutant allele forward 5′TTGGTGGTCGAATGGGCAGGTA3′ and reverse 5′CGCGCTGTTCTCCTCTTCCTCA3′; *Nod2* wild-type allele forward 5′ACAGAGATGCCGACACCATACTG3′ and reverse 5′TGGAGAAGGTTGAAGAGCAGAGTC3′; *Nod2* mutant allele forward 5′TGACTGTGGCTAATGTCCTTTGTG3′ and reverse 5′TTCTATCGCCTTCTTGACGAGTTC3′.

### Ethics statement

All protocols involving animal experiments were approved and carried out in accordance with the ethical guidelines of Ribeirão Preto Medical School, University of São Paulo, Brazil (License number: 047/2007). All mice were handled with regard for alleviation of suffering. Analgesics were not used as treatment in mice after sepsis induction, due to their possible interference via the production of inflammatory mediators. The mice were housed in the animal facility of the Department of Pharmacology in this School under controlled light/dark cycle, temperature and humidity. Maximum of five mice per polysulfone cages (32 cm×20 cm×21 cm) was allowed. Food and water were provided *ad libitum*.

After sepsis induction, mice showed piloerection, crusty exudates around their eyes, reduced locomotion and altered breath frequency. These events were worsened as the mice approached death. Mice (except for survival experiments) were euthanized in different time points by overdose of ketamine and xylazine (>100/10 mg/kg, s.c., União Química, BR) followed by cervical dislocation. For survival experiments, we monitored mice with CLP-induced sepsis at each 12 h for 10 days. At this time, mice that show signs of imminent death (i.e. inability to maintain upright position/ataxia/tremor and/or agonal breathing) were euthanized by ketamine/xylazine overdose followed by cervical dislocation. At the end of the survival experiment, live mice were also euthanized by ketamine/xylazine overdose followed by cervical dislocation.

### Sepsis model

CLP-induced sepsis was performed as previously described with slight modification [20]. Briefly, mice were anesthetized with ketamine/xylazine (100/10 mg/kg, i.p., União Química, BR), and the cecum was exposed and punctured using 30, 21 or 18G needle (BD, USA) to induce non-severe (NS), moderate (M) or severe (S) sepsis, respectively. The animals received 1 mL of saline subcutaneously after this procedure to avoid dehydration and returned to the same cages they were previously housed in, with water and food *ad libitum*.

Sepsis was also induced by polymicrobial inoculation. Bacteria were isolated from the cecal microbiota of WT mice as previously described [21]. Optical density of bacterial suspension was adjusted to 0.380, which was previously determined to be equivalent to approximately 1.4×10^8^ bacteria per 0.1 mL. This volume was homogenized with an equal volume of 10% gelatin solution (Synth, BR) and intraperitoneally (i.p.) administered to WT, *Nod1*- or *Nod2*-deficient mice to mimic the bacterial infection induced by CLP.

### Neutrophil recruitment to the peritoneal cavity

Neutrophil recruitment was assessed 6, 12 or 24 h after sepsis induction as indicated in the legends. The animals were euthanized, and the cells in the peritoneal cavity were harvested by the introduction of 1.5 mL of PBS containing 1 mM EDTA. Total and differential cell counts were performed as previously described [22].

### Determination of cytokine and chemokine levels in the peritoneal lavage

The animals were euthanized 6, 12 or 24 h after sepsis induction and peritoneal lavage was performed. Cytokines and chemokines concentrations were determined using a standard sandwich ELISA. CXCL1, CXCL2, IL-6 and TNF-α were measured using commercially available antibodies following the procedures supplied by the manufacturer (R&D Systems, USA).

### LPS, LTA and CXCL2 stimulation in vivo

Lipopolysaccharide (LPS) (200 µg/cavity, Difco, USA), Lipoteichoic acid (LTA) (30 µg/cavity, Sigma-Aldrich, USA), CXCL2 (30 ng/cavity, R&D Systems, USA) or saline were i.p. administered to WT and *Tlr2*-, *Tlr4*- and *MyD88-*deficient mice as indicated in the figure legend. Peritoneal lavage was harvested 6 h after stimulation to determine the neutrophil recruitment into the peritoneal cavity.

### Chemotaxis assay

Neutrophils were isolated from bone marrow of WT and *MyD88*-deficient mice as previously described [7]. Isolated neutrophils (5×10^4^/well) were incubated with CXCL1, CXCL2 (10 ng/mL) or RPMI in a Boyden chamber as previously described by others [23].

### Adoptive transfer of neutrophils from WT and *MyD88*-deficient mice into WT mice

Adoptive neutrophil transfer was performed as previously described with modifications [7]. Neutrophils isolated from bone marrow of WT and *MyD88*-deficient mice were stained with 0.5 or 5 µM CFSE, respectively, in order to differentiate the two lineages of neutrophils. These neutrophils were mixed and i.v. administered (5×10^6^/mouse) to a WT mouse 2 h before non-severe sepsis induction. Peritoneal lavage was harvested 6 h after CLP surgery. The neutrophils from the peritoneal lavage were stained with anti-GR-1 (BD Biosciences, USA) and the CFSE-stained neutrophils were analyzed using a GR-1 positive gate. Samples were acquired on a FACS Calibur flow cytometer (BD Biosciences, USA) and analyzed using FCS Express V3 software.

### Resident peritoneal cell culture

Resident peritoneal cells were harvested by washing the peritoneal cavity of mice with PBS containing 1 mM EDTA. The cells were incubated with LPS (1 µg/mL), LTA (10 µg/mL) or RPMI for 24 h. Supernatant CXCL2 and TNF-α levels were determined by ELISA.

### Transfer of resident peritoneal cells from *MyD88*-deficient or WT mice into *MyD88*-deficient mice

Cells harvested from the peritoneal cavity of WT or *MyD88*-deficient mice were administered i.p. (5×10^6^/cavity) into *MyD88*-deficient mice. The CLP surgery was performed 2 h after the transfer of the cells into the animal. The peritoneal lavage was harvested 6 h after sepsis induction to evaluate CXCL2 levels and neutrophil recruitment.

### Bacterial count in the peritoneal lavage and blood

The bacterial count was determined as previously described [24]. Briefly, mice were euthanized 6, 12 or 24 h after sepsis induction. After harvesting the blood and peritoneal lavage using sterile PBS, aliquots of serial dilutions of these samples were plated on Muller-Hinton agar plates (OXOID, UK) and incubated at 37°C. CFUs were analyzed 18 h after the incubation.

### Expression of *Nod1* and *Nod2*

Peripheral blood was harvested from *naïve* WT mice 6 h after sepsis induction, and mononuclear cells and neutrophils were isolated using Percoll, as previously described [7]. RNA extraction was performed using TRIzol (Life Technologies, USA) and following the manufacturer’s instructions. Reverse transcription of total RNA to cDNA was carried out by performing a reverse transcription reaction (IMPROM II, Promega, USA). Real-time PCR was performed using specific primers for *Nod1* and *Nod2* and the housekeeping gene *Gapdh*. Reactions were conducted on ViiA7 using the SYBR-green FAST fluorescence system (both from Applied Biosystems, USA). The data were analyzed with the 2- ΔΔCt method. Primer pairs for the *Nod1*, *Nod2* and *Gapdh* were as follows: *Gapdh*, forward 5′CATCTTCTTGTGCAGTGCCA3′ and reverse 5′CGGCCAAATCCGTTCAC3′; *Nod1*, forward 5′CATGATCCAGCAAAGCAATA3′ and reverse 5′CCATACCCTTCTTCTCATCC3′; *Nod2*, forward 5′AACTGTCCAACAATGGCATC3′ and reverse 5′TTCCCTCGAAGCCAAACCT3′.

### Myeloperoxidase (MPO) assay

Lungs were harvested from mice at 6, 12 or 24 h and processed as previously described [6].

### Statistical analysis

The data expressing survival rates are expressed as percentage of surviving animals and the Mantel-Cox log-rank test was used to determine differences between survival curves. Bacterial counts are expressed as medians and the remaining data are expressed as mean ± standard error of the mean (SEM) of values obtained from the experiments. The strain effect was analyzed by multifactorial ANOVA with factors being strain and time (6 and 24 h data expressed in the same graph). The data expressed in one timepoint were analyzed by ANOVA followed by Dunnett’s test or by unpaired *t test* in the case of comparison between only two groups. A *P* value of 0.05 or less was considered significant. Unless otherwise specified in the figure legend, the sample size for each experiment was *n* = 5 mice per group as previously standardized for these protocols in our laboratory. All of the statistical analyzes were performed using GraphPad Prism version 5.00 for Windows (GraphPad Software, USA).

## Results

### *Nod1* and *Nod2* expression is not altered during CLP-induced sepsis

To evaluate whether *Nod1* or *Nod2* mRNAs were systemically expressed by leukocytes during severe sepsis, we isolated peripheral blood mononuclear cells (PBMCs) and neutrophils from *naïve* and septic mice 6 h after CLP. RT-qPCR analysis revealed constitutive expression of *Nod1* and *Nod2* in PBMCs and circulating neutrophils (Figure S1a and b). Importantly, no alteration was observed in the *Nod1* or *Nod2* expression levels 6 h after polymicrobial sepsis induction.

### *Nod1* and *Nod2* are not crucial to the outcome of polymicrobial sepsis

**Figure 1:**
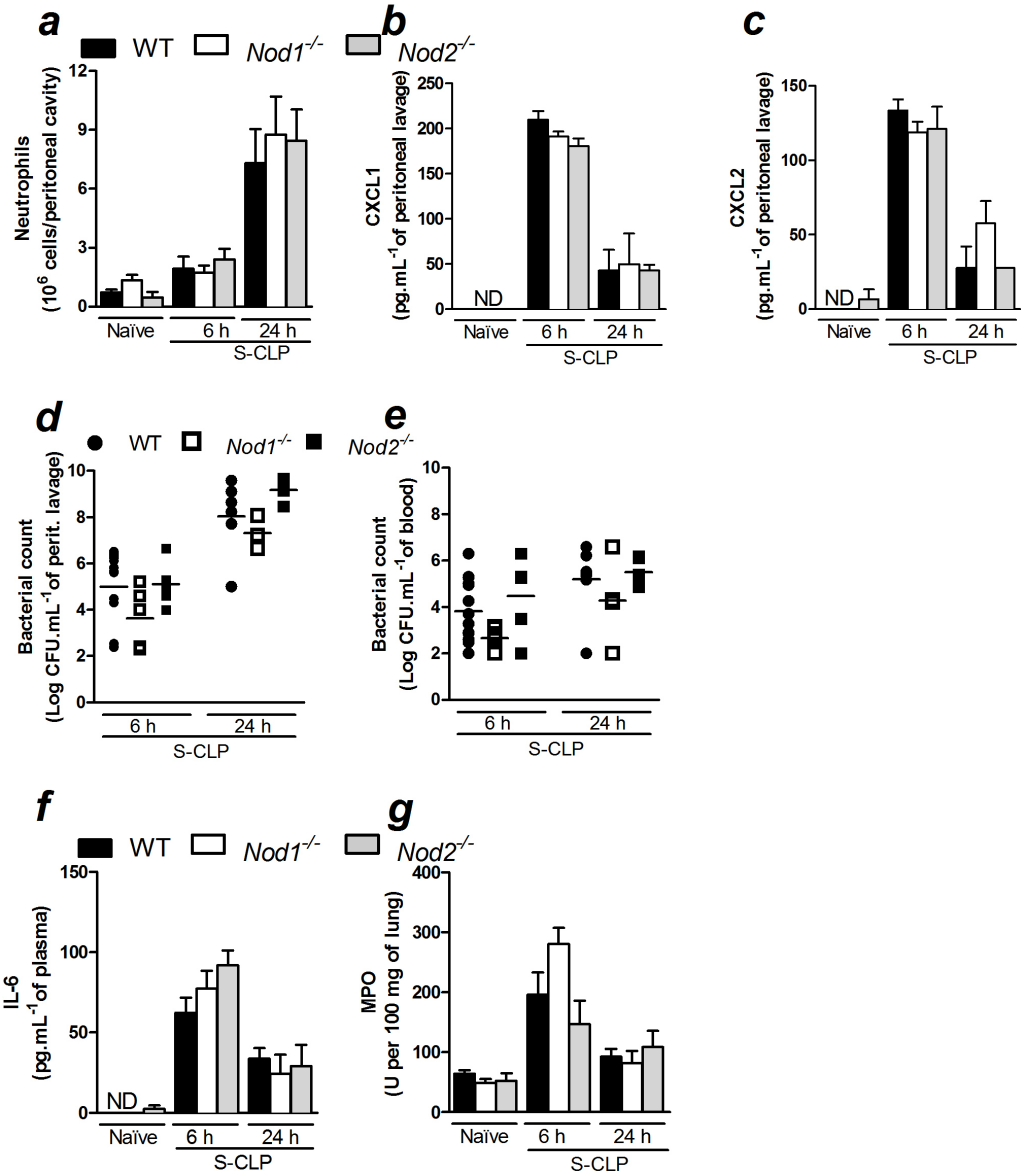
Nod1 and Nod2 are not crucial for the inflammatory response during severe polymicrobial sepsis. Six or 24*Nod1*- and *Nod2*-deficient mice (WT, *Nod1* ^−/−^ and *Nod2* ^−/−^, respectively) underwent CLP-induced severe sepsis they were assessed for: a) neutrophil recruitment to the peritoneal cavity; b) CXCL1 and c) CXCL2 levels in the peritoneal lavage, as measured by ELISA; d) bacterial count in the peritoneal lavage and e) blood; f) IL-6 levels in plasma; g) lung MPO activity. The data were analyzed by multifactorial ANOVA and are expressed as the mean ± SEM in *a*, *b*, *c*, *f* and *g* and as median in *d* and *e.* The graphs represent the mean of the results of two or three independent experiments. *n = 3* to *5* per experiment; ND = not detected.

Nod1 ligands have been described to induce neutrophil recruitment *in vivo* [18]. Additionally, our group has extensively shown the importance of neutrophil recruitment for the recovery from sepsis [3]. Taking this information into consideration, we evaluated whether Nod1 or Nod2 is involved in the recruitment of neutrophils to the site of infection during polymicrobial sepsis. Neutrophil recruitment into the peritoneal cavity was assessed in *Nod1*- and *Nod2*-deficient mice following CLP-induced sepsis. Unexpectedly, neutrophil recruitment was similar in WT, *Nod1*- and *Nod2*-deficient mice 6 (Figure S2a) and 12 h after non-severe sepsis induced by CLP (data not shown). Likewise, these mice showed similar neutrophil recruitment 6, 12 and 24 h after CLP-induced severe sepsis (Figure S3a and Figure 1a). We also measured CXCL1 and CXCL2 levels at the site of infection. In agreement with the neutrophil recruitment data, CXCL1 and CXCL2 levels were similar in the three experimental groups in all tested time points after severe sepsis induction (Figures 1b, 1c, S3b and S3c). Moreover, CXCL2 levels were also similar 6 hours after non-severe sepsis induction (Figure S2b). The deficiencies of *Nod1* and *Nod2* in these mice were confirmed where, in contrast to WT, *Nod1*- and *Nod2*-deficient mice were not able to recruit neutrophils in response to the i.p. administration of FK565 or MDP, respectively (Figure S4a and S4b). This set of results indicates that neither Nod1 nor Nod2 is involved in the release of neutrophil chemotactic mediators and, consequently, in the recruitment of neutrophils during CLP-induced sepsis.

In agreement with the results of the neutrophil recruitment assay, the bacterial loads in the peritoneal cavity and the blood were also similar in WT, *Nod1*- and *Nod2*-deficient mice 6 h after non-severe (Figure S2c) and 6, 12 or 24 h after severe sepsis induced by CLP (Figures 1d, 1e and S3d). These data suggest that neither Nod1 nor Nod2 plays a significant role in the control of the bacterial burden during CLP-induced sepsis.

**Figure 2:**
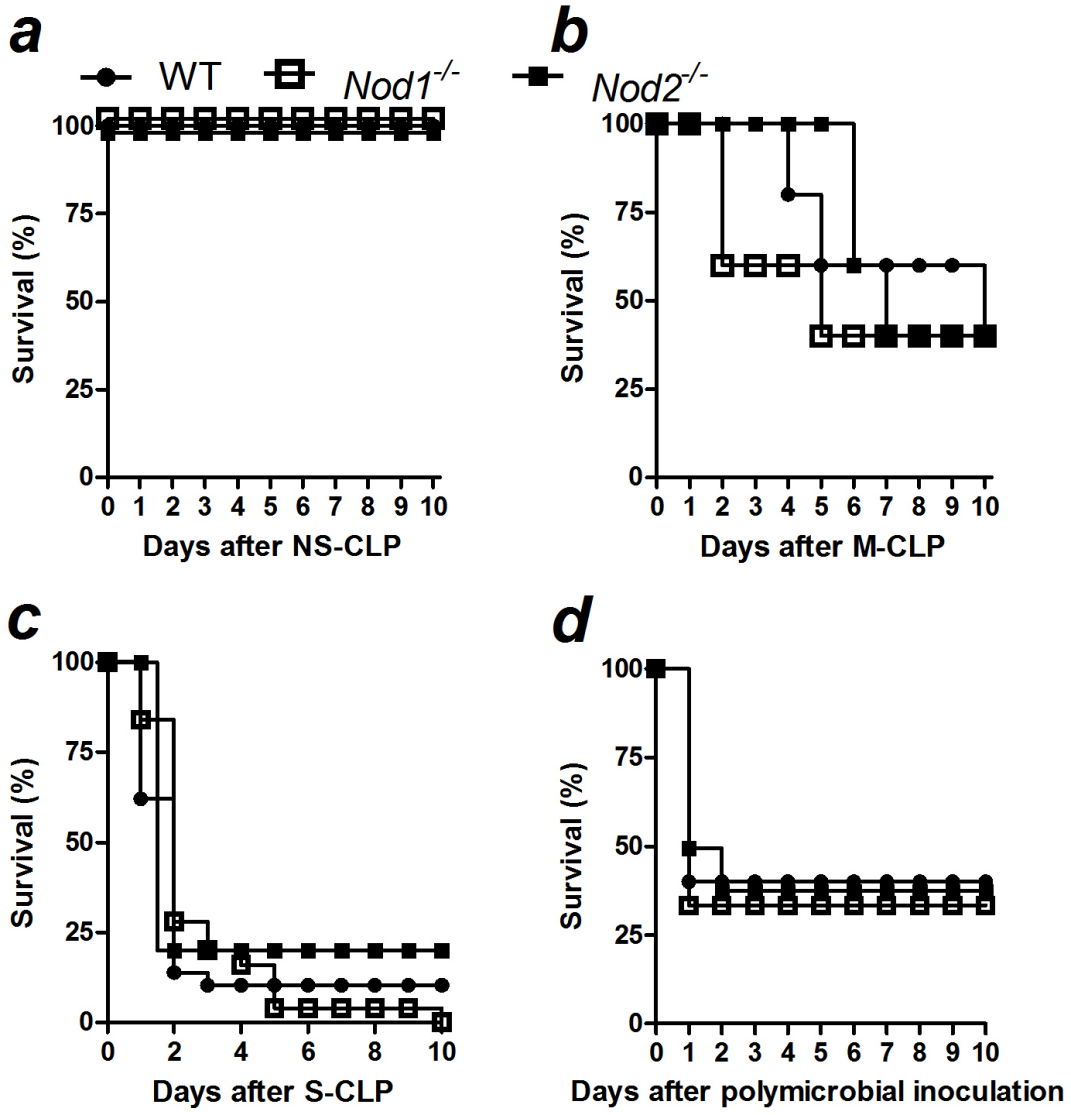
Nod1 and Nod2 are not essential for the resistance to polymicrobial sepsis. Sepsis was induced in WT, *Nod1*- and *Nod2*-deficient mice (WT, *Nod1* ^−/−^ and *Nod2* ^−/−^, respectively) using CLP model. The survival curve was observed up to 10 days after the induction of a) non-severe (NS), b) moderate (M), c) severe (S) sepsis or d) after the intraperitoneal inoculation of 1.4×10^6^ cecal-isolated bacteria per mice. The results are expressed as percentage of survival and were analyzed by the Mantel-Cox log-rank test. The graphs represent the mean of the results of two or three independent experiments. *N = 5 to 6* per experiment.

In addition, we demonstrated that plasma-derived IL-6 levels in *Nod1*- and *Nod2*-deficient or WT mice were similar 6, 12 or 24 h after severe sepsis (Figure 1f and S3e), as well as 6 h after non-severe sepsis induction (Figure S2d). We also evaluated whether Nod1 or Nod2 is involved in the sequestration of neutrophils in the lung, thus contributing to pulmonary dysfunction during sepsis. WT, *Nod1*- and *Nod2*-deficient mice contained similar amounts of neutrophils sequestration in the lung in all times and both severities tested, as estimated by MPO activity (Figures 1g, S2e and S3f). In agreement with the previous data, WT, *Nod1*- and *Nod2*-deficient mice showed similar survival rates in CLP-induced non-severe, moderate and severe sepsis (Figures 2a, b and c). To confirm that the absence of a phenotype in *Nod1*- and *Nod2*-deficient mice during polymicrobial sepsis was not the consequence of a microbiotic variation in the knockout mice, we inoculated the cecal bacteria isolated from WT mice into the peritoneal cavities of WT, *Nod1-* and *Nod2*-deficient mice. The *Nod1*- and *Nod2*-deficient mice showed survival rates similar to WT mice after peritoneal polymicrobial inoculation (Figure 2d).

### Deficiency in both *Nod1* and *Nod2* does not interfere with the outcome of CLP-induced sepsis

**Figure 3:**
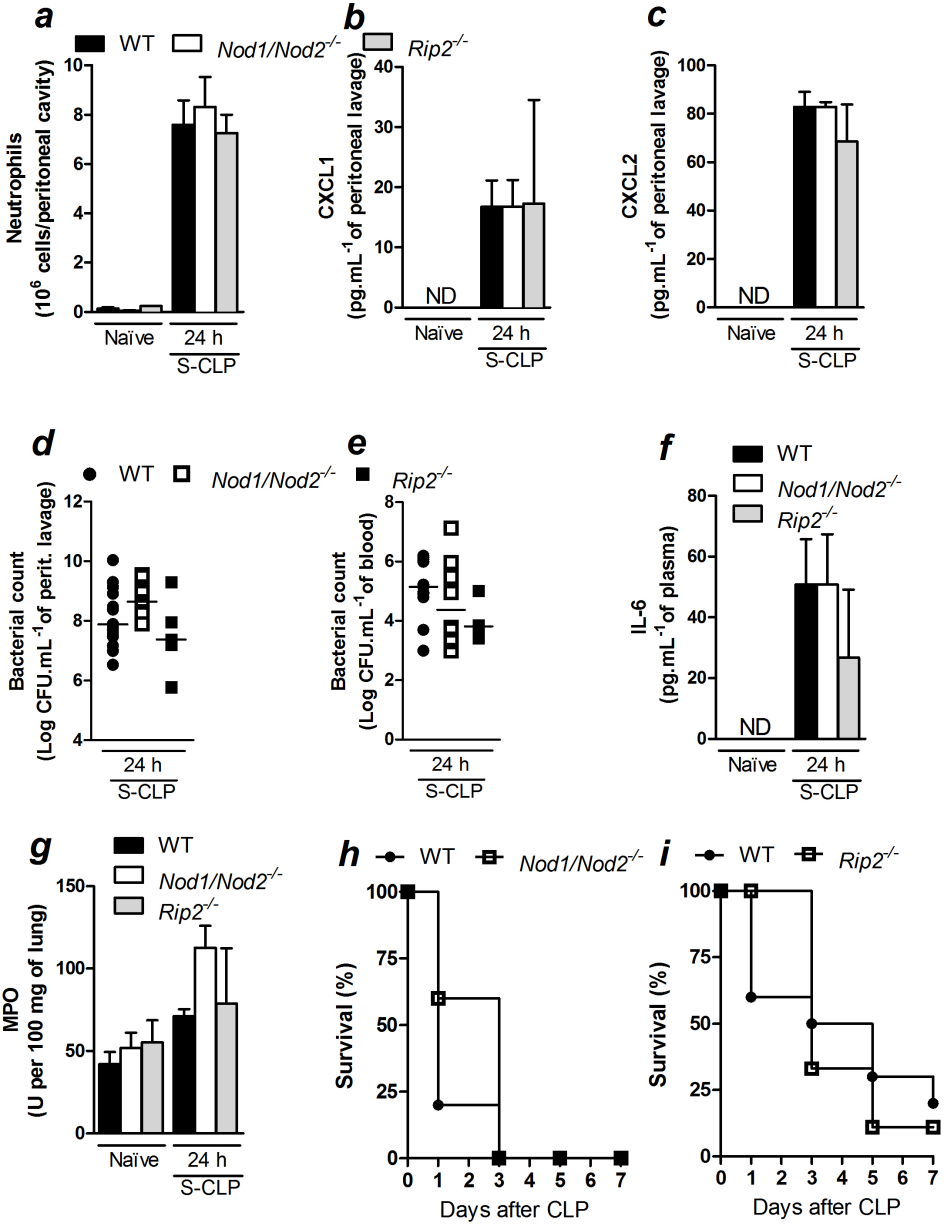
The additive response of Nod1 and Nod2 is not essential to the inflammatory response during severe polymicrobial sepsis. At 24*Nod1/Nod2-* and *Rip2*-deficient mice (*Nod1/Nod2* ^−/−^ and *Rip2* ^−/−^, respectively) underwent CLP they were assessed for: a) neutrophil recruitment to the peritoneal cavity; b) CXCL1 and c) CXCL2 levels in the peritoneal lavage as measured by ELISA; d) bacterial count in peritoneal lavage and e) blood; f) IL-6 levels in plasma; g) lung MPO activity; h) survival of WT and *Nod1/Nod2-*deficient mice and i) *Rip2*-deficient mice post-CLP-induced severe sepsis. The data are expressed as the mean ± SEM in *a*, *b*, *c*, *f* and *g;* median in *d* and *e;* and as a percentage of survival in *h* and *i.* The data in *a*, *b, c, d, e, f* and *g* were analyzed by multifactorial ANOVA and the data in *h* and *i* were analyzed by Mantel-Cox log-rank test. The results are representative of at least two independent experiments. *n = 5 to 8; ND = *not detected.

Because both Nod1 and Nod2 sense bacterial cell wall components and signal via Rip2 kinase [14], we investigated whether there was a possible synergy between Nod1 and Nod2 during severe polymicrobial sepsis. Thus, we tested whether the lack of phenotypic differences in *Nod1* and *Nod2* single deficient mice was due to a functional redundancy. We induced sepsis and measured inflammatory readouts 24 h after CLP in mice that were doubly deficient in *Nod1* and *Nod2* (*Nod1/Nod2*) and in mice that were deficient in *Rip2*. Similar to *Nod1-* and *Nod2-* single deficient mice, neutrophil recruitment to the peritoneal cavity 24 h after CLP surgery was not different among WT, *Nod1/Nod2*- and *Rip2*-deficient mice (Figure 3a). Moreover, there were no differences in CXCL1 or CXCL2 production at the site of infection in WT, *Nod1/Nod2*- and *Rip2*-deficient mice (Figures 3b and 3c). As a consequence, there were no differences observed in the peritoneal bacterial loads (Figure 3d) or in the blood bacterial loads (Figure 3e) in WT, *Nod1/Nod2*- or *Rip2*-deficient mice. Collectively, these data indicate that an additive response of Nod1 and Nod2 is not essential in neutrophil recruitment or bacterial control during CLP-induced severe sepsis.

We also analyzed the systemic pro-inflammatory cytokine production of these mice. As observed in *Nod1*- and *Nod2*-deficient mice, *Nod1/Nod2*- or *Rip2*-deficient mice that experienced CLP-induced severe sepsis produced similar systemic levels of IL-6 (Figure 3f) and TNF-α (WT, *Nod1/Nod2*- and *Rip2-deficient* mice, respectively: *Naïve* = 3.01±1.3; 4.0±1.2; 3.5±1.6 pg.mL^−1^ of plasma and after CLP = 25.3±5.2; 25.3±7.3; 21.8±1.2 pg.mL^−1^ of plasma, n = 5). Similar levels of MPO activity were observed in the lungs of WT, *Nod1/Nod2*- and *Rip2*-deficient mice 24 h after CLP-induced severe sepsis (Figure 3g), further confirming that these pathogen sensors are not relevant to neutrophil sequestration in the lung. Moreover, the survival rates for *Nod1/Nod2*- (Figure 3h) and *Rip2*-deficient mice (Figure 3i) were similar to WT mice that underwent CLP surgery for the duration of the experiment.

*Nod1*-, *Nod2*- and *Rip2*-deficient mice were co-housed with WT mice to generate similar microbiota. However, there was still no major role for Nod1 or Nod2 in neutrophil recruitment (Figure S5a), chemokine production (Figure S5b and S5c), bacterial load (Figure S5d and S5e) or IL-6 levels (Figure S5f) after CLP surgery. Taken together, these results suggest that Nod1 and Nod2 do not play a role in survival, chemokine production, neutrophil recruitment to the infectious site, or sequestration to the lungs after CLP-induced severe sepsis.

### *MyD88* is crucial to the establishment of the local inflammatory response and resistance to CLP-induced sepsis

**Figure 4:**
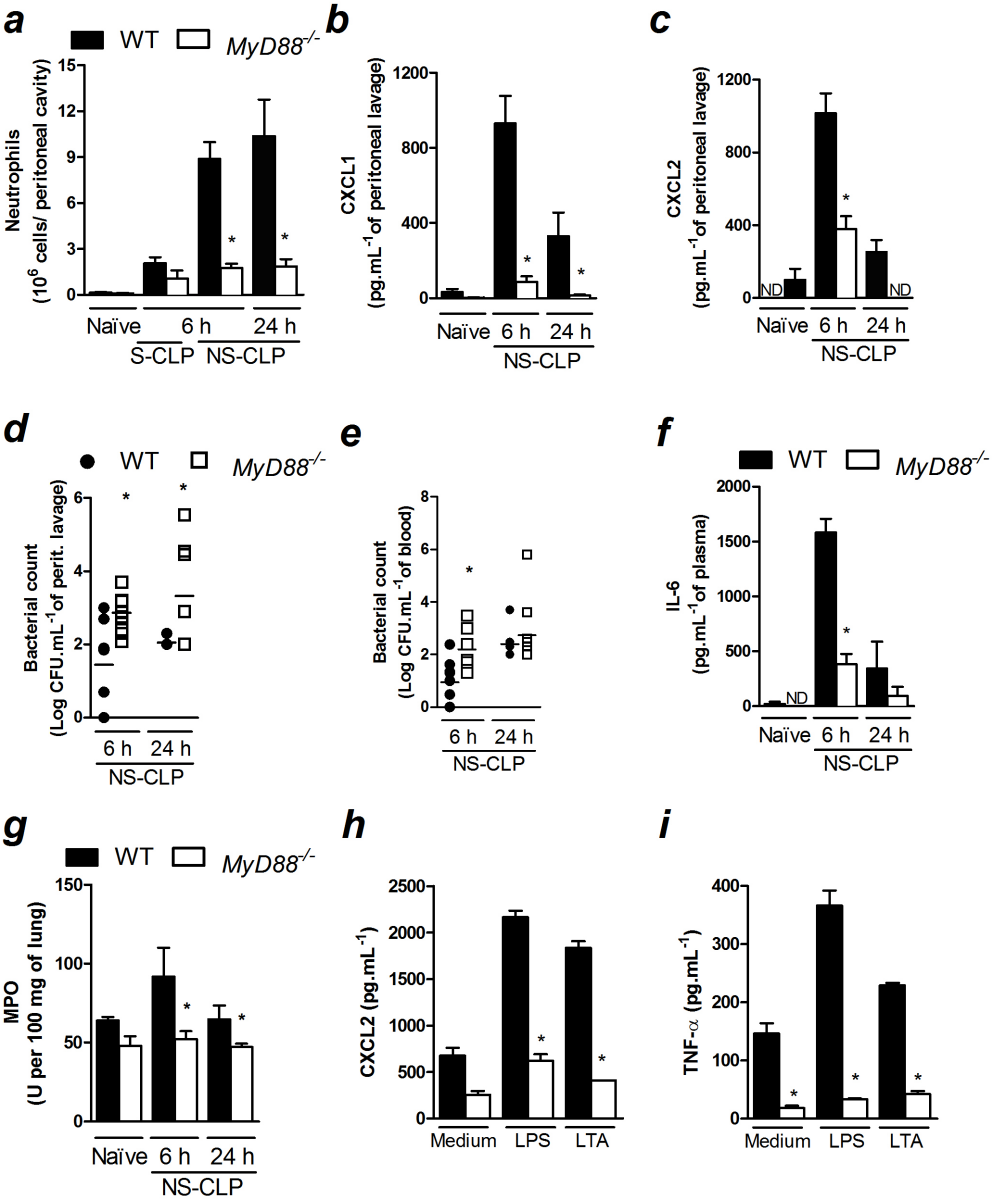
MyD88 is crucial for the resolution of non-severe sepsis. a–g) Non-severe (NS) and severe (S) sepsis were induced by CLP in WT and *MyD88*-deficient mice and 6 or 24 h after sepsis induction, the following were assessed: a) neutrophil recruitment to the peritoneal cavity; b) CXCL1 and c) CXCL2 levels in the peritoneal lavage determined by ELISA; d) bacterial count in the peritoneal lavage and e) blood; f) IL-6 levels in the plasma, as measured by ELISA and g) lung MPO activity. h) Peritoneal macrophages were stimulated with LPS (1 µg/mL) or LTA (10 µg/mL) for 24 h and CXCL2 and i) TNF-α were measured in the supernatant by ELISA. These data are expressed as the mean ± SEM in *a, b*, *c, f*, *g, h* and *i* and median in *d* and *e*. The data were analyzed by multifactorial ANOVA followed by unpaired *t test*. The graphs are representative of one to four independent experiments. *n = *4 to 10*; ND = *not detected.*P<0.05.

Because we showed that Nod1/Nod2 signaling was not indispensable for the production of chemokines and neutrophil recruitment to the infection site during polymicrobial sepsis, we investigated the importance of MyD88, the main adaptor protein of the TLR family, in these events. We found that neutrophil recruitment was markedly reduced in *MyD88*-deficient mice 6 and 24 h after non-severe sepsis induction (Figure 4a). In agreement with this observation, *MyD88*-deficient mice produced significantly less neutrophil chemokines CXCL1 and CXCL2 at the infection site after non-severe sepsis induction (Figures 4b and c). As expected, increased bacterial load in the peritoneal cavities and blood of *MyD88*-deficient mice were observed after non-severe sepsis induction (Figures 4d and e). Next, we also observed that *MyD88*-deficient mice had a reduction in IL-6 in the plasma 6 h after non-severe sepsis induction (Figures 4f). Due to the low plasma levels of IL-6 24 h after non-severe sepsis induction, we were not able to find significant difference between WT and *MyD88*-deficient mice. Corroborating the reduced systemic IL-6 levels, we also observed a reduction in neutrophil accumulation into the lungs of *MyD88*-deficient mice 6 and 24 h after non-severe sepsis induced by CLP (Figure 4g). Furthermore, the deleterious effect of the absence of MyD88 was also observed in mice under severe sepsis. Despite the reduction in the neutrophil recruitment in *MyD88*-deficient mice 6 h after severe sepsis induction was not significant (Figure 4a), these mice showed diminished CXCL2 levels at the infection site under this severity of sepsis (Figure S6a). In addition, *MyD88*-deficient mice also showed increased bacterial load in the peritoneal cavity and blood (Figure S6b), as well as reduced IL-6 levels in the plasma 6 h after severe sepsis induction (Figure S6c). Mice under severe sepsis were only evaluated 6 h after CLP because all mice were dead within 24 h. Furthermore, we investigated the ability of cytokine/chemokine production by peritoneal-elicited macrophages under *in vitro* stimulation. *MyD88*-deficient macrophages stimulated with LPS or LTA produced less CXCL2 and TNF-α than macrophages harvested from WT mice (Figures 4h and 4i). These data suggest that MyD88 is crucial for the release of chemotactic mediators during CLP-induced sepsis.

**Figure 5:**
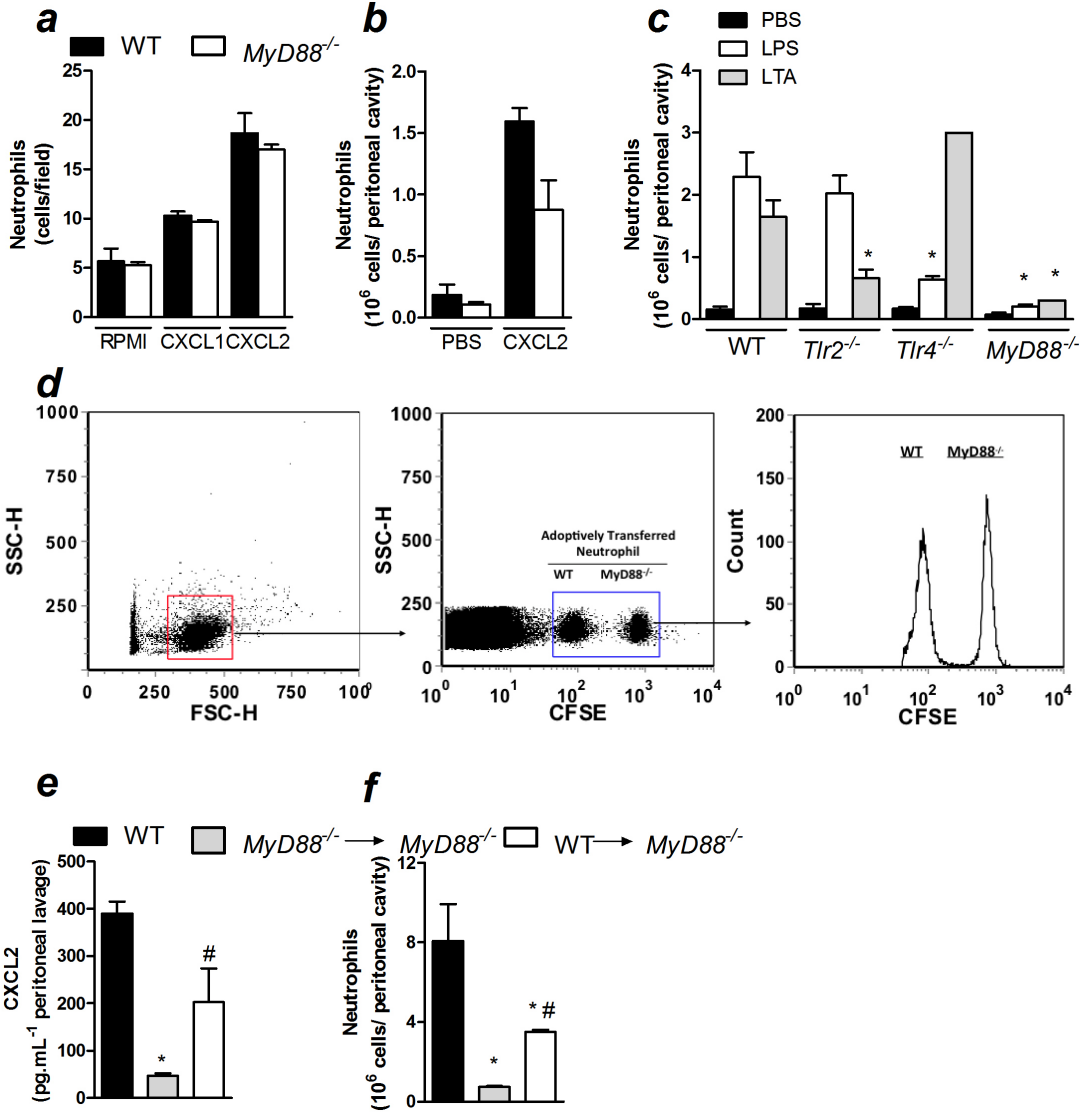
MyD88 is crucial for the establishment of the inflammatory response during polymicrobial sepsis. a) Bone marrow-isolated neutrophils (5×10^4^/well) from WT or *MyD88*-deficient mice (*MyD88* ^−/−^) were stimulated by CXCL1 or CXCL2 (10 ng/mL) in a Boyden chamber to measure chemotaxis. b) Neutrophil recruitment to the peritoneal cavity 6 h after an i.p. injection of CXCL2 (30 ng/cavity). c) Neutrophil recruitment to the peritoneal cavity 6 h after an i.p. injection of LPS (200 µg/cavity) or LTA (30 µg/cavity) in WT, *Tlr2*-, *Tlr4*- and *MyD88*-deficient mice. d) Neutrophils from the bone marrow of WT or *MyD88-*deficient mice were stained with different concentrations of CFSE and administered (5×10^6^/mouse; i.v.) into WT mice 2 h before non-severe (NS) sepsis induction by CLP. Cells in the peritoneal lavage were harvested 6 h after CLP surgery and analyzed by flow cytometry. For e–f, resident peritoneal cells from WT and *MyD88-*deficient mice were harvested and transferred (5×10^6^/intraperitoneal cavity) to *MyD88*-deficient mice 30 minutes before CLP surgery. e) CXCL2 was measured by ELISA in the peritoneal lavage at 6 h after CLP surgery, as was the f) neutrophil recruitment to the peritoneal cavity. The data are expressed as the mean ± SEM and were analyzed by unpaired *t test*. *P<0.05 compared to WT and ^#^ *P*<0.05 compared to *MyD88^−/−^→MyD88^−/−^*; n = 5.

To test whether the reduction in the neutrophil recruitment in *MyD88*-deficient septic mice is also due to a deficiency in the chemotactic ability of these cells, we assessed neutrophil chemotaxis in response to CXCL1 or CXCL2 *in vitro*. In addition, we evaluated neutrophil recruitment in response to CXCL2 *in vivo* in non-septic WT and *MyD88*-deficient mice. There were no differences in neutrophil migration in either assay (Figures 5a and 5b). Thus, the results indicated that the reduction in neutrophil recruitment in *MyD88*-deficient mice was not due to a reduction in the chemotactic ability of the neutrophils. Moreover, the fact that the *in vivo* recruitment of neutrophils induced by CXCL2 administration in non-septic animals was not reduced in *MyD88*-deficient mice suggests that the mechanics of neutrophil-to-endothelium adhesion are not compromised in these animals. Altogether, the results suggest that the reduction in neutrophil recruitment in *MyD88*-deficient mice during sepsis is a consequence of the impairment in the production of chemotactic mediators. It was also observed that the ligands for TLR2 and TLR4 did not induce neutrophil recruitment *in vivo* in their respective gene-deficient mice or in *MyD88*-deficient mice (Figure 5c). These results suggest that the compromised neutrophil recruitment in *MyD88*-deficient mice is associated with the absence of signaling pathways downstream of MyD88 that may be involved in the production of neutrophil chemotactic mediators, rather than an impaired recognition of the ligands. Moreover, although MyD88 is also downstream of the IL-1R and IL-18 pathways, neutrophil recruitment 6 h after non-severe sepsis induction was similar between *IL-18*-deficient mice treated with IL-1Ra and control mice (Figure S7). Therefore, the phenotype observed in *MyD88-*deficient mice is likely because of the absence of TLR signaling.

To further confirm that a deficiency in MyD88 impairs the establishment of the local inflammatory response, neutrophils from WT and *MyD88*-deficient mice were stained using different CFSE concentrations and tracked after being injected intravenously into WT mice 2 h before sepsis induction. Similar numbers of WT and *MyD88*-deficient neutrophils were found in the peritoneal lavage of WT mice after sepsis induction (Figure 5d). Moreover, *MyD88*-deficient resident peritoneal cells adoptively transferred into *MyD88*-deficient mice maintained a reduced level of CXCL2 production (Figure 5e) and impaired neutrophil recruitment to the peritoneal cavity (Figure 5f). However, the adoptive transfer of WT resident cells into *MyD88*-deficient mice before the induction of sepsis resulted in the increase of local levels of CXCL2. Additionally, there was an increase in neutrophil recruitment to the peritoneal cavity during CLP-induced sepsis.

**Figure 6:**
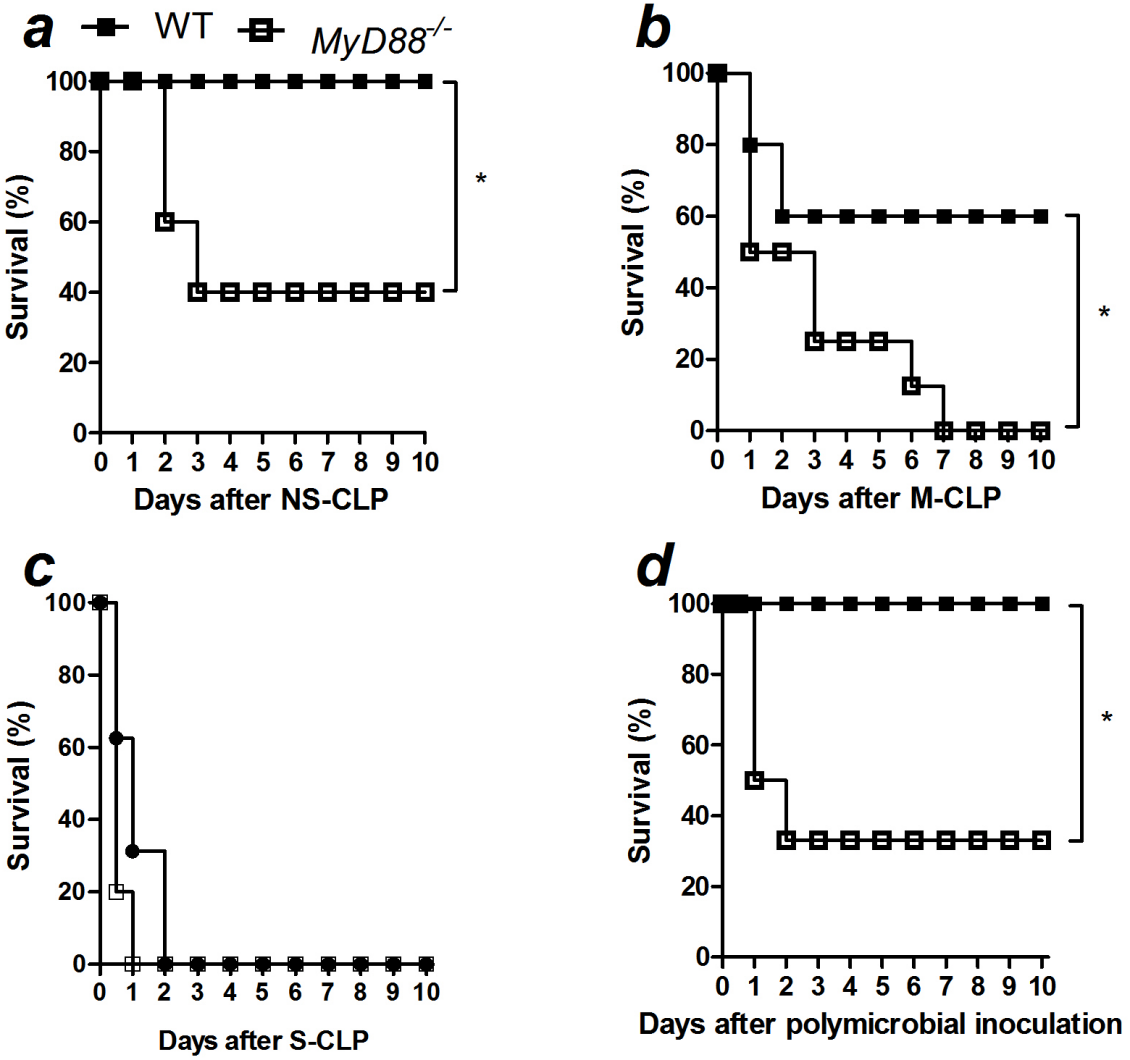
MyD88 is essential for the resistance to polymicrobial sepsis. Sepsis was induced in WT and *MyD88*-deficient mice (WT, *MyD88* ^−/−^, respectively) using CLP model. The survival curve was observed up to 10 days after the induction of a) non-severe (NS), b) moderate (M), c) severe (S) sepsis or d) after the intraperitoneal inoculation of 1.4×10^6^ cecal-isolated bacteria per mice. The results are expressed as percentage of survival and were analyzed by Mantel-Cox log-rank test. The graphs represent one of one to two independent experiments. *N = 5 to 6* per experiment. *P<0.05.

Finally, we evaluated the survival of *MyD88*-deficient mice. As expected, *MyD88*-deficient mice were more susceptible to non-severe and moderate sepsis (Figures 6a and b). Although no significant difference was found between WT and *MyD88*-deficient mice, the former mice died earlier than WT under severe sepsis (Figure 6c). Moreover, *MyD88*-deficient mice were more susceptible to polymicrobial infection induced by cecal bacteria isolated from WT (Figure 6d), suggesting that the observed effect in *MyD88*-deficient mice was not due to differences in the microbiota between both strains of mice. These data support the hypothesis that *MyD88*-deficient mice fail to produce chemotactic mediators, resulting in impaired neutrophil recruitment to the infection site, increased bacterial loads and an increased susceptibility to pathology in CLP-induced sepsis.

## Discussion

During mammalian pathogen recognition the immune response is mainly activated by TLRs and NLRs [4]. Based on the known roles of Nod1 and Nod2, members of the NLR family, in the induction of an immune response during infection [25], we investigated whether these receptors were involved in the physiopathology of sepsis. We demonstrated that, under our experimental conditions, neither Nod1 nor Nod2 was essential to the onset of inflammation at the infection site, analyzed by chemokine production and neutrophil recruitment, during polymicrobial sepsis. By contrast, the adaptor protein MyD88, which is required for TLR activation, is crucial for the establishment of a local chemotactic response and neutrophil recruitment to the site of infection, which consequently determines the outcome of polymicrobial sepsis.

In agreement with previous reports showing the expression of *Nod1* and *Nod2* in human monocytes, the THP-1 mononuclear cell line and human neutrophils [26]–[28], we showed that *Nod1* and *Nod2* are constitutively expressed in mouse blood mononuclear cells and neutrophils. However, their expression was not altered during CLP-induced sepsis. It has been previously demonstrated that the expression of *Nod1* and *Nod2* is induced in osteoblasts by stimulation with heat-killed *Salmonella spp.* and *Staphylococcus spp.*, while *Nod2* expression is also upregulated by TNF-α and IFN-γ in the bronchial epithelial cell line BEAS-2B [29], [30]. In this context, it is unusual that the expression of *Nod1* and *Nod2* was not altered after CLP-induced sepsis.

It has been previously reported that Nod1 ligands induce chemokine production and neutrophil recruitment *in vivo* [18], [31]. Moreover, we have shown that *Legionella pneumophila*, an intracellular bacterium, recruits neutrophils to the site of infection in a Nod1- and Nod2-dependent manner [32]. Similarly, the immune response to several pathogens such as *Listeria monocytogenes*, *Helicobacter pylori* and *Chlamydophila pneumoniae* is triggered by both Nod1 and Nod2 activation and the consequent recruitment of the downstream signaling protein Rip2 [33], [34]. However, we demonstrated in this study that the production of chemokines and neutrophil recruitment after CLP were not altered in *Nod1*- or *Nod2*-deficient mice nor were they altered in *Nod1/Nod2* double-deficient or *Rip2*-deficient mice. It has been shown that the number of neutrophils that are recruited to an infection site is inversely proportional to the bacterial load [22]. In this study, we demonstrated that Nod1 and Nod2 did not function in neutrophil recruitment, and as consequence, we observed no difference in the bacterial load at the site of infection in *Nod1-* or *Nod2-*deficient mice following neither non-severe nor severe sepsis induced by CLP. The local levels of IL-6 and TNF-α were also similar in WT, *Nod1*- and *Nod2*-deficient mice 24 h after severe sepsis induction (data not shown). Furthermore, an efficient local inflammatory response is needed to prevent the bacteria from disseminating from the site of infection into the blood [35]. We observed no difference in the systemic bacterial loads in *Nod1*-, *Nod2*-, *Nod1/Nod2*- and *Rip2*-deficient mice in comparison to WT mice during sepsis.

Sepsis is a complex syndrome, whose outcome depends on the correct activation of different systems, including the immune system. When the immune response is successfully organized to clear the pathogen, the organ damage is not critical, avoiding the host death. Based on that, we would be tempted to suggest that Nod1 and Nod2 do not play a major role in polymicrobial sepsis, since the absence of them did not alter the survival. However, we cannot exclude an unessential role of these receptors in any of the multiple responses involved during the septic episode, which we did not focus on, in this study. It has been recently demonstrated that Nod2 mediates the production of IL-10, which enhances the production of C5a, contributing to an increased mortality rate after CLP-induced sepsis [36]. Here we did not evaluate the role of Nod1 or Nod2 on the eicosanoid and complement production and we cannot explain the apparent discrepancy regarding the involvement of Nod2 in sepsis. However, given the importance of the microbiota as an inducer of infection in CLP-induced sepsis, we believe that differences in the microbiota could lead to activation of divergent signaling pathways and could culminate in contrasting phenotypes. In this work, we demonstrate that in our experimental conditions, differences in the microbiota of WT and *Nod1-, Nod2-* or *Rip2-*deficient mice are not responsible for the absence of a phenotype in CLP-induced sepsis.

Additionally, the systemic inflammatory response during a severe septic episode is characterized by high serum levels of cytokines and chemokines and by neutrophil accumulation in different organs, which leads to dysfunction [35], [37]. These events are closely associated with the presence of components or the whole bacteria in the blood [37]. It has been previously shown that the direct activation of Nod1 by the systemic administration of its ligand induces IL-6 production in wild-type mice [17]. Moreover, the cited study demonstrated that the systemic administration of Nod1 ligands induced shock and organ dysfunction in experimental animals. In this study, we did not detect different levels of circulating IL-6 or neutrophil accumulation in the lungs during sepsis in *Nod1*-, *Nod2*-, *Nod1/Nod2*- and *Rip2-*deficient mice compared to WT septic mice. However, we showed that Nod1 and Nod2 specific ligands, FK565 and MDP, respectively, induced neutrophil migration in mice. We believe that the administration of Nod1 and Nod2 specific ligands activate the receptors and their signaling pathway leading to the observed effects. By contrast, the whole bacteria observed during CLP-induced sepsis do not activate Nod1 and Nod2, or does it in a weak intensity. The majority of the bacteria that translocate from the cecum to the peritoneal cavity may be extracellular microorganisms that may primarily activate the extracellular domains of the TLRs rather than inducing the activation of the intracellular receptors Nod1 and Nod2.

Indeed, our group has previously reported that TLR2, 4 and 9 play a deleterious role in CLP-induced severe sepsis [7], [8], [38]. It is believed that the activation of many TLRs during the polymicrobial challenge contributes to an overwhelming of the inflammatory response that is observed in sepsis and may lead to high mortality rates. However, here we demonstrate that the abrogation of most TLR signaling, as assessed in *MyD88-*deficient mice, leads to increased susceptibility to sepsis because of the inability to establish a local inflammatory response. Similarly, others have also shown the reduction of an inflammatory response in *MyD88*-deficient mice during polymicrobial sepsis [10], [11], [13]. We have demonstrated that the absence of MyD88 signaling impairs the establishment of the local inflammatory response induced by CLP, which results in a decline in neutrophil recruitment to the site of infection. This decline results in an enhancement of bacterial dissemination into the circulation and a reduction in the survival of the mice. Indeed, *MyD88*-deficient mice lack the production of neutrophil chemokines (CXCL1, CXCL2) at the infection site. It may also be hypothesized that a MyD88 deficiency does not affect the ability of the neutrophils to respond to chemotactic stimuli because *MyD88*-deficient mice had a similar amount of neutrophil recruitment to WT mice in response to the intraperitoneal CXCL2 stimulus. Moreover, the adoptive transfer of resident peritoneal cells from WT to *MyD88*-deficient mice induced an increase in CXCL2 production and neutrophil recruitment. These data are reinforced by Gais *et al.;* they have shown that the peritoneal inflammatory response was fully established in mice with selective expression of MyD88 in myeloid cells during polymicrobial sepsis. In addition, the mentioned study also shows that the reduced neutrophil accumulation into lungs was restored in mice expressing MyD88 in myeloid cells [39]. This supports that the reduction in neutrophil accumulation in the lungs of *MyD88*-deficient mice described here, results from the reduced systemic inflammation, despite the increased bacterial load in them. Together, these data therefore indicate that MyD88-dependent signaling is essential for the recognition of pathogens and triggering of the immune response in polymicrobial sepsis. Based on our findings, we cannot exclude the roles of Nod1 and Nod2 in sepsis in the absence of major signaling pathways induced by TLR. In this context, the remaining levels of produced chemokines and recruited neutrophils in *MyD88*-deficient mice could result in the activation of Nod1 and Nod2, which was not enough to control the infection and resolve the septic episode.

To the best of our knowledge, our findings are the first to describe that *MyD88*-deficient mice are impaired in the control of bacterial growth during polymicrobial sepsis. In accordance with our data, Peck-Palmer *et al.* demonstrated that *MyD88*-deficient mice have higher mortality rates than WT mice [12]. By contrast, there are reports showing that *MyD88*-deficient mice are more resistant to polymicrobial sepsis due to reduced systemic inflammation [10], [11], [13]. It is known that both reduced systemic inflammatory response and organ damage are beneficial in sepsis and it is a rational explanation for the reduced mortality in *MyD88*-deficient mice in the cited studies. However, the control of the infection is another feature that is just as important as the systemic inflammatory response, and in our experimental conditions, the bacterial load control in mice lacking MyD88 was decisive for their outcome, even though these mice had reduced accumulation of neutrophils in the lung, as evidenced by MPO assay. Even, the intensity of the infecting stimuli does not seem to be the explanation because we observed that *MyD88*-deficient mice had higher and faster mortality when undertaken to non-severe, moderate and severe sepsis.

In conclusion, our data indicate that Nod1 and Nod2 do not play a major role in the resolution of polymicrobial sepsis in our experimental conditions. By contrast, we demonstrated that MyD88-dependent signaling is crucial for sepsis because the removal of this molecule completely abrogated the induction of a local chemotactic response, culminating in a higher susceptibility to CLP-induced sepsis.

## Supporting Information

Figure S1 **The expression of** ***Nod1*** **and** ***Nod2*** **is not altered in PBMCs or neutrophils after CLP-induced severe sepsis.** a) mRNA expression was evaluated 6 h after CLP surgery in naïve or septic mice. PBMCs and b) circulating neutrophils were isolated, and *Nod1* and *Nod2* expression levels were determined using qPCR. The data are expressed as the mean ± SEM of at least two independent experiments and were analyzed by unpaired *t test*. *n* = 3 to 5 per experiment.
(TIF)

Figure S2 **Nod1 and Nod2 are not crucial for the inflammatory response during non-severe polymicrobial sepsis.** Six hours after WT, *Nod1*- and *Nod2*-deficient mice (WT, *Nod1* ^−/−^ and *Nod2* ^−/−^, respectively) underwent CLP-induced non-severe (NS) sepsis they were assessed for: a) neutrophil recruitment to the peritoneal cavity; b) CXCL2 levels in the peritoneal lavage, as measured by ELISA; c) bacterial count in the peritoneal lavage and blood; d) IL-6 levels in plasma and e) lung MPO activity. The data were analyzed by ANOVA followed by Dunnett’s test and are expressed as the mean ± SEM in *a*, *b*, *d* and *e,* and as median in *c.* The graphs are representative of one or two independent experiments. *n = 3* to *5* per experiment.
(TIF)

Figure S3 **Nod1 and Nod2 are not crucial for the inflammatory response during severe polymicrobial sepsis.** Twelve hours after WT, *Nod1*- and *Nod2*-deficient mice (WT, *Nod1* ^−/−^ and *Nod2* ^−/−^, respectively) underwent CLP-induced severe (S) sepsis they were assessed for: a) neutrophil recruitment to the peritoneal cavity; b) CXCL1 and c) CXCL2 levels in the peritoneal lavage, as measured by ELISA; d) bacterial count in the peritoneal lavage and blood; e) IL-6 levels in plasma and f) lung MPO activity. The data were analyzed by ANOVA followed by Dunnett’s test and are expressed as the mean ± SEM in *a*, *b*, *c*, *e,* and *f* and as median in *d.* The graphs represent the mean of one or three independent experiments. *n = 3* to *5* per experiment.
(TIF)

Figure S4 **FK565 and MDP do not induce neutrophil recruitment in** ***Nod1*** **- and** ***Nod2*** **-deficient mice, respectively.** a) Neutrophil migration in the peritoneal cavity was evaluated in WT and *Nod1*-deficient mice 6 h after i.p. administration of PBS or 1 mg/kg FK565 (kindly provided by Astellas Pharm Inc., Japan). b) Neutrophil migration was also evaluated in WT and *Nod2*-deficient mice 6 h after i.p. administration with 300 µg/cavity of muramyldipeptide (Sigma-Aldrich, USA). The data are expressed as the mean ± SEM and were analyzed by unpaired *t test*, *P<0.05, *n = 5*.
(TIF)

Figure S5 **The absence of a phenotype in** ***Nod1*** **-,** ***Nod2*** **- and** ***Rip2*** **-deficient mice is not due to different microbiota.** WT mice were co-housed with *Nod1*-, *Nod2*- or *Rip2*-deficient mice (*Nod1* ^−/−^, *Nod2* ^−/−^ or *Rip2* ^−/−^, respectively) or were housed separately for 4 weeks before CLP. After 24 h of severe (S) sepsis induction, we assessed the following: a) neutrophil recruitment to the peritoneal cavity; b) CXCL1 and c) CXCL2 levels in the peritoneal lavage, as measured by ELISA; d) bacterial count in the peritoneal lavage and e) blood; and f) IL-6 levels in the plasma, as measured by ELISA. The data are expressed as the mean ± SEM in *a*, *b*, *c* and *f*, and as the median in *d* and *e.* The data were analyzed by ANOVA followed by Dunnett’s test. *n = 5 to 8; ND = *not detected.
(TIF)

Figure S6 **MyD88 is essential for the inflammatory response during severe polymicrobial sepsis.** Six hours after WT and *MyD88*-deficient mice (WT, *MyD88* ^−/−^, respectively) underwent CLP-induced severe (S) sepsis they were assessed for: a) CXCL2 levels in the peritoneal cavity; b) bacterial count in the peritoneal lavage and blood and c) IL-6 levels in plasma. The data were analyzed by unpaired *t* test and are expressed as mean ± SEM in *a* and *c* and as median in *b.* The graphs are representative of three independent experiments. *n = 5*.
(TIF)

Figure S7 **IL-1 and IL-18 signaling are not involved in neutrophil migration during non-severe CLP-induced sepsis.** Neutrophil recruitment to the peritoneal cavity was assessed 6 h after mild sepsis induction in WT (Balb/c) and *Il18*-deficient mice (*Il18* ^−/−^). IL-1Ra (i.v. 30 mg/Kg) was administered 15 min prior to CLP surgery. The data are expressed as the mean ± SEM and were analyzed by ANOVA, followed by Dunnett’s Test using the WT as the control. *n = 3* to *5* per experiment.
(TIF)
