# Supplementary figures and images for: MyD88-, but Not Nod1- and/or Nod2-Deficient Mice, Show Increased Susceptibility to Polymicrobial Sepsis due to Impaired Local Inflammatory Response
Source: PLoS One. 2014 Aug 1;9(8):e103734. doi: 10.1371/journal.pone.0103734 (PMC4118952; doi:10.1371/journal.pone.0103734)

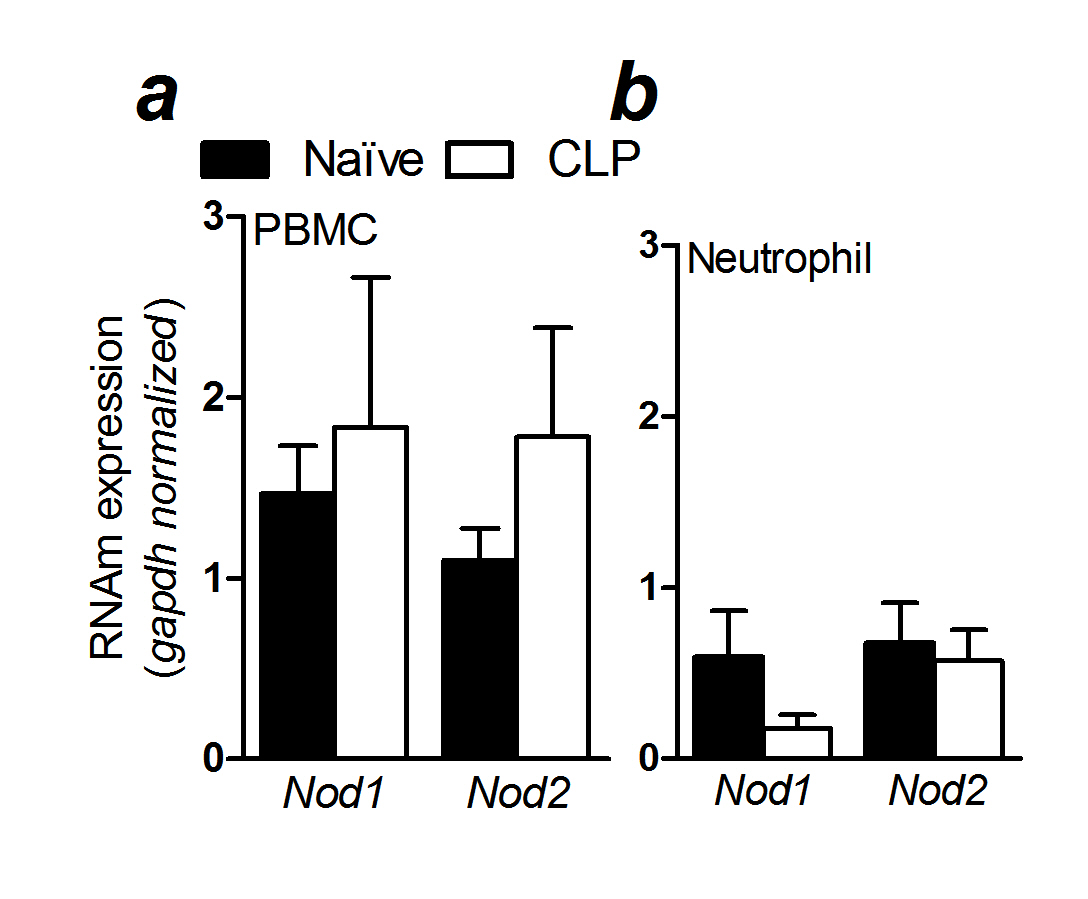

Supplement: Figure S1 — The expression of Nod1 and Nod2 is not altered in PBMCs or neutrophils after CLP-induced severe sepsis. a) mRNA expression was evaluated 6 h after CLP surgery in naïve or septic mice. PBMCs and b) circulating neutrophils were isolated, and Nod1 and Nod2 expression levels were determined using qPCR. The data are expressed as the mean ± SEM of at least two independent experiments and were analyzed by unpaired t test. n = 3 to 5 per experiment. (TIF) [file pone.0103734.s001.tif]

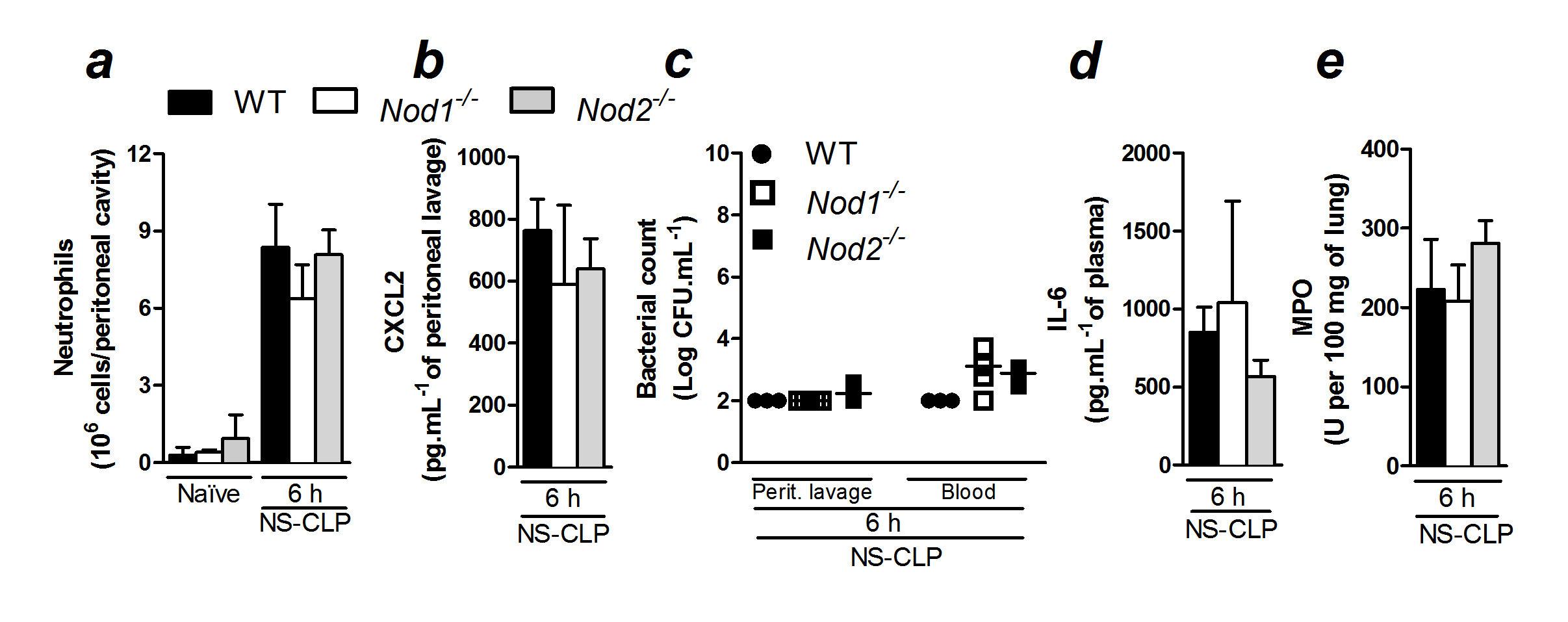

Supplement: Figure S2 — Nod1 and Nod2 are not crucial for the inflammatory response during non-severe polymicrobial sepsis. Six hours after WT, Nod1- and Nod2-deficient mice (WT, Nod1 −/− and Nod2 −/−, respectively) underwent CLP-induced non-severe (NS) sepsis they were assessed for: a) neutrophil recruitment to the peritoneal cavity; b) CXCL2 levels in the peritoneal lavage, as measured by ELISA; c) bacterial count in the peritoneal lavage and blood; d) IL-6 levels in plasma and e) lung MPO activity. The data were analyzed by ANOVA followed by Dunnett’s test and are expressed as the mean ± SEM in a, b, d and e, and as median in c. The graphs are representative of one or two independent experiments. n = 3 to 5 per experiment. (TIF) [file pone.0103734.s002.tif]

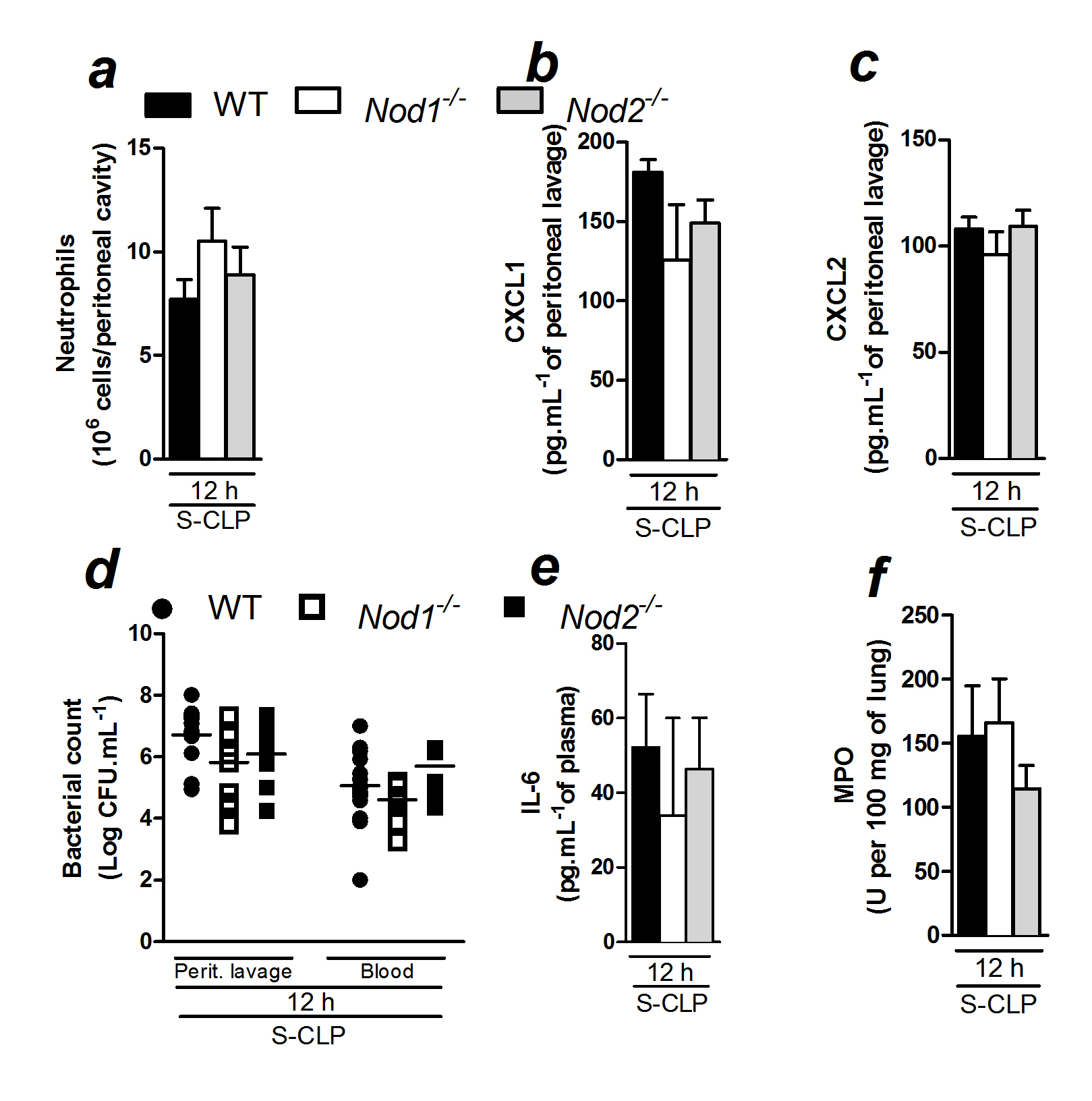

Supplement: Figure S3 — Nod1 and Nod2 are not crucial for the inflammatory response during severe polymicrobial sepsis. Twelve hours after WT, Nod1- and Nod2-deficient mice (WT, Nod1 −/− and Nod2 −/−, respectively) underwent CLP-induced severe (S) sepsis they were assessed for: a) neutrophil recruitment to the peritoneal cavity; b) CXCL1 and c) CXCL2 levels in the peritoneal lavage, as measured by ELISA; d) bacterial count in the peritoneal lavage and blood; e) IL-6 levels in plasma and f) lung MPO activity. The data were analyzed by ANOVA followed by Dunnett’s test and are expressed as the mean ± SEM in a, b, c, e, and f and as median in d. The graphs represent the mean of one or three independent experiments. n = 3 to 5 per experiment. (TIF) [file pone.0103734.s003.tif]

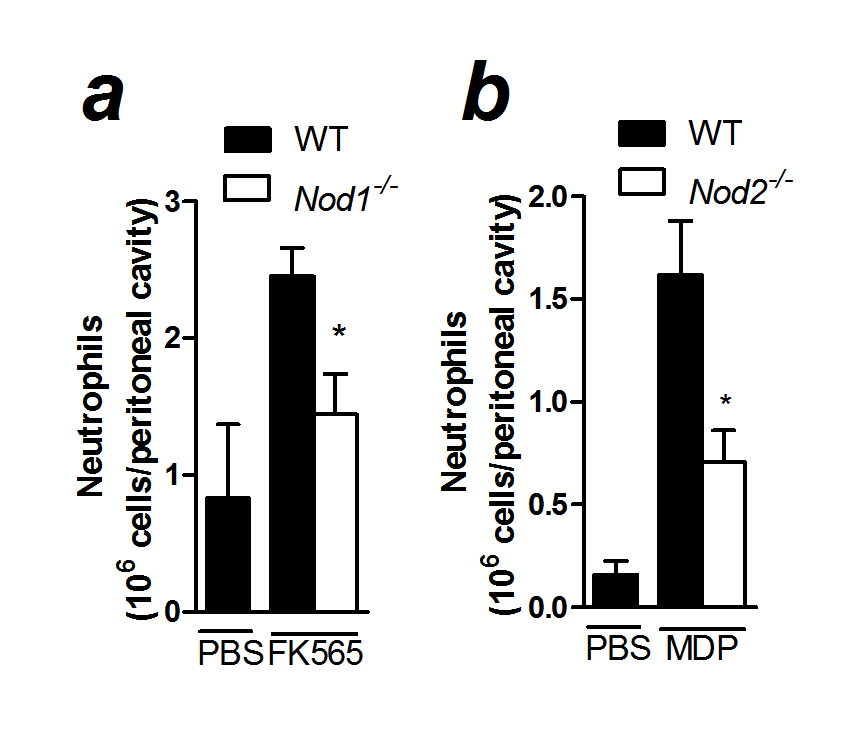

Supplement: Figure S4 — FK565 and MDP do not induce neutrophil recruitment in Nod1 - and Nod2 -deficient mice, respectively. a) Neutrophil migration in the peritoneal cavity was evaluated in WT and Nod1-deficient mice 6 h after i.p. administration of PBS or 1 mg/kg FK565 (kindly provided by Astellas Pharm Inc., Japan). b) Neutrophil migration was also evaluated in WT and Nod2-deficient mice 6 h after i.p. administration with 300 µg/cavity of muramyldipeptide (Sigma-Aldrich, USA). The data are expressed as the mean ± SEM and were analyzed by unpaired t test, *P<0.05, n = 5. (TIF) [file pone.0103734.s004.tif]

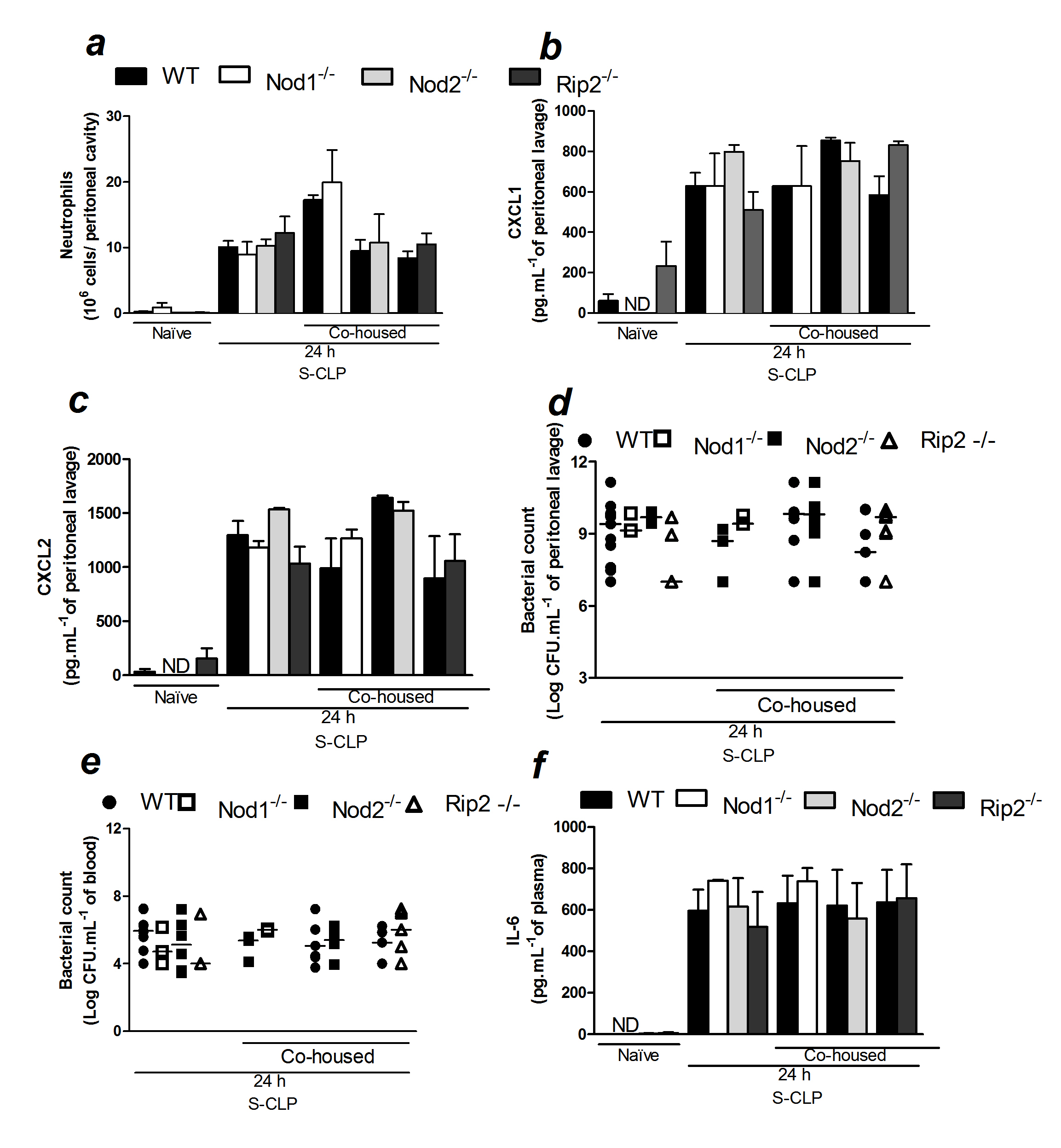

Supplement: Figure S5 — The absence of a phenotype in Nod1 -, Nod2 - and Rip2 -deficient mice is not due to different microbiota. WT mice were co-housed with Nod1-, Nod2- or Rip2-deficient mice (Nod1 −/−, Nod2 −/− or Rip2 −/−, respectively) or were housed separately for 4 weeks before CLP. After 24 h of severe (S) sepsis induction, we assessed the following: a) neutrophil recruitment to the peritoneal cavity; b) CXCL1 and c) CXCL2 levels in the peritoneal lavage, as measured by ELISA; d) bacterial count in the peritoneal lavage and e) blood; and f) IL-6 levels in the plasma, as measured by ELISA. The data are expressed as the mean ± SEM in a, b, c and f, and as the median in d and e. The data were analyzed by ANOVA followed by Dunnett’s test. n = 5 to 8; ND = not detected. (TIF) [file pone.0103734.s005.tif]

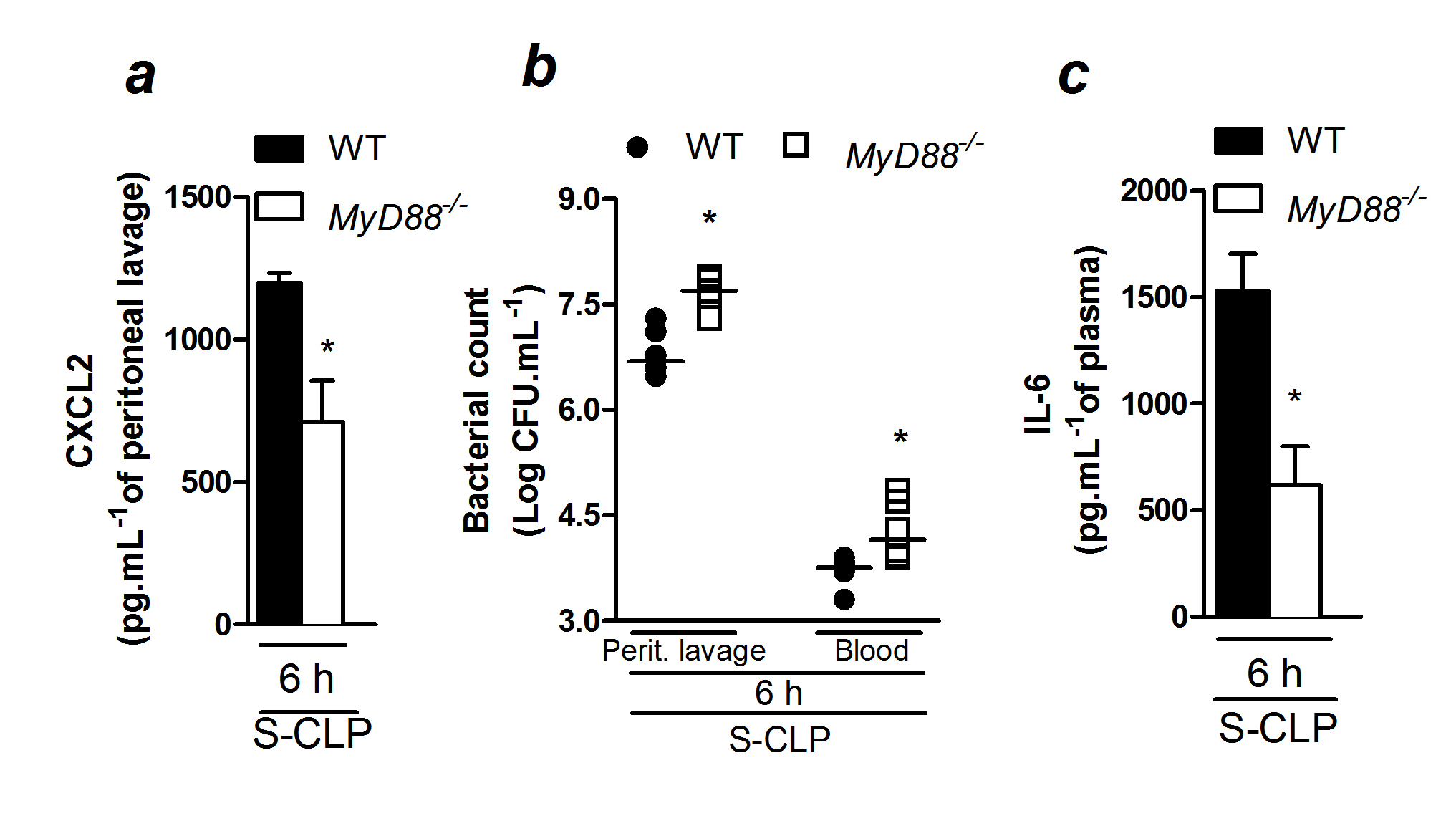

Supplement: Figure S6 — MyD88 is essential for the inflammatory response during severe polymicrobial sepsis. Six hours after WT and MyD88-deficient mice (WT, MyD88 −/−, respectively) underwent CLP-induced severe (S) sepsis they were assessed for: a) CXCL2 levels in the peritoneal cavity; b) bacterial count in the peritoneal lavage and blood and c) IL-6 levels in plasma. The data were analyzed by unpaired t test and are expressed as mean ± SEM in a and c and as median in b. The graphs are representative of three independent experiments. n = 5. (TIF) [file pone.0103734.s006.tif]

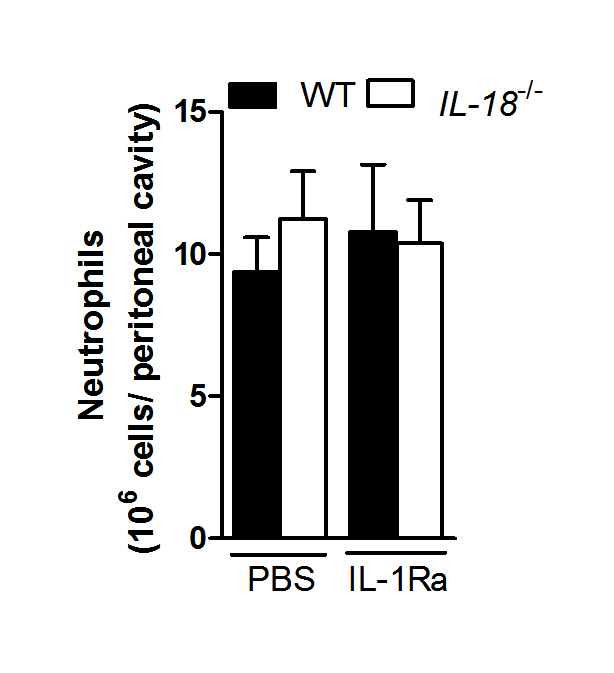

Supplement: Figure S7 — IL-1 and IL-18 signaling are not involved in neutrophil migration during non-severe CLP-induced sepsis. Neutrophil recruitment to the peritoneal cavity was assessed 6 h after mild sepsis induction in WT (Balb/c) and Il18-deficient mice (Il18 −/−). IL-1Ra (i.v. 30 mg/Kg) was administered 15 min prior to CLP surgery. The data are expressed as the mean ± SEM and were analyzed by ANOVA, followed by Dunnett’s Test using the WT as the control. n = 3 to 5 per experiment. (TIF) [file pone.0103734.s007.tif]
